# Diagnostic Yield of Routine Pericardial Fluid Cultures and Clinical Predictors of Bacterial Pericarditis

**DOI:** 10.3390/diagnostics16152328

**Published:** 2026-07-25

**Authors:** Amir Orlev, Alon Ze’ev Yefet, Tali Bdolah-Abram, Elad Asher, Yonit Wiener-Well

**Affiliations:** 1 Jesselson Integrated Heart Center, The Eisenberg R&D Authority, Shaare Zedek Medical Center, Faculty of Medicine, The Hebrew University of Jerusalem, Jerusalem 9103102, Israel; easher@szmc.org.il; 2 Faculty of Medicine, The Hebrew University of Jerusalem, Jerusalem 9112000, Israel; alonzee.yefet@mail.huji.ac.il; 3 The Center for Medical Education, Faculty of Medicine, The Hebrew University of Jerusalem, Jerusalem 9112001, Israel; 4 Infectious Disease Unit, The Eisenberg R&D Authority, Shaare Zedek Medical Center, Faculty of Medicine, The Hebrew University of Jerusalem, Jerusalem 9112102, Israel; yonitw@szmc.org.il

**Keywords:** pericardial effusion, pericardiocentesis, bacterial pericarditis, pericardial fluid culture, infection

## Abstract

**Background**: Pericardial effusion has a broad differential diagnosis, whereas bacterial pericarditis is rare. Despite this, routine culturing of pericardial fluid is frequently performed, and its diagnostic yield remains uncertain. **Methods**: We conducted a retrospective study of all pericardial fluid cultures obtained at a tertiary medical center between January 2016 and May 2024. Cultures were classified as true positive if a recognized bacterial pathogen was isolated or if it was deemed clinically pathogenic by an infectious disease’s specialist at the medical record. Contaminants were defined according to CDC criteria. Clinical, laboratory, and imaging variables were analyzed using univariate testing and multivariable logistic regression. **Results**: Among 198 eligible pericardial fluid cultures, 8 (4%) yielded true bacterial pathogens, whereas 43 (23%) grew contaminants and 147 (73%) remained sterile. Thus, contaminant growth occurred more than five times as frequently as that of true pathogens. The most frequent pathogens were Staphylococcus aureus and Streptococcus species. No demographic features, presenting symptoms, or imaging findings were associated with culture positivity. In multivariable analysis, concurrent bacteremia (odds ratio [OR] 17.49; *p* = 0.002) and higher peripheral neutrophil percentage (OR 1.20; *p* = 0.012) independently predicted bacterial pericarditis. In models incorporating pericardial fluid cell counts, a lower mononuclear cell percentage was also independently associated with infection (OR 0.91; *p* = 0.03). **Conclusions**: Bacterial pericarditis is uncommon, and routine pericardial fluid cultures, of all pericardial fluid specimens, have a low diagnostic yield with substantial contamination. Laboratory markers, particularly bacteremia and neutrophilia, may help identify patients most likely to benefit from culture. A selective, laboratory-guided approach may improve diagnostic efficiency and reduce unnecessary testing.

## 1. Introduction

Pericardial effusion is the accumulation of excess fluid (>50 mL) within the pericardial sac. Although a small volume of serous fluid is normally present and facilitates cardiac motion, pathologic accumulation may develop through increased fluid production, impaired lymphatic drainage, inflammation, hemorrhage, or malignant involvement. The differential diagnosis is broad and includes malignancy, autoimmune disease, renal failure, trauma, post-procedural states, and infection [1,2,3,4]. In high-income countries, acute idiopathic or presumed viral pericarditis is among the most common inflammatory causes, whereas tuberculosis remains an important cause in endemic regions and among selected high-risk groups [5,6,7].

Clinical suspicion for pericardial effusion is based on the integration of symptoms, physical examination, electrocardiography, and imaging. Patients may present with dyspnea, chest pain, fever, hypotension, or nonspecific constitutional symptoms; however, many effusions are detected incidentally during imaging performed for other indications. Classical findings such as muffled heart sounds, low QRS (ventricular depolarization complex on electrocardiography) voltage, electrical alternans, or cardiomegaly on chest radiography are neither uniformly present nor specific. Echocardiography remains the principal diagnostic modality because it enables estimation of effusion size, assessment of chamber collapse, and evaluation for tamponade physiology. Computed tomography and cardiac magnetic resonance imaging may provide additional information regarding loculation, pericardial thickening, adjacent infection, malignancy, or complex postoperative anatomy [8,9,10].

Pericardiocentesis is not routinely indicated for every patient with pericardial effusion. Accepted indications include cardiac tamponade, suspected purulent or tuberculous pericarditis, suspected malignant effusion, and large symptomatic effusions requiring diagnostic clarification or therapeutic drainage [11]. Purely diagnostic pericardiocentesis in uncomplicated acute pericarditis has historically demonstrated a low yield, particularly when the clinical presentation is typical and there are no high-risk features [12]. Therefore, diagnostic testing of pericardial fluid should be guided by the pretest probability of clinically actionable disease [11].

When pericardial drainage is performed, routine fluid evaluation commonly includes cell count with differential, Gram stain, bacterial culture, cytology, glucose, protein, and lactate dehydrogenase. Additional tests are generally reserved for specific clinical contexts, such as triglyceride measurement for suspected chylopericardium, acid-fast staining and mycobacterial culture for suspected tuberculosis, and molecular assays for viral pathogens or Mycobacterium tuberculosis when conventional methods are expected to have limited sensitivity [7,13]. Despite these recommendations, bacterial cultures are often sent routinely whenever pericardial fluid is obtained, even when the clinical suspicion for bacterial pericarditis is low [14].

Infectious causes account for only a minority of pericardial effusions, and contemporary series have reported bacterial pericarditis in a small proportion of patients undergoing pericardiocentesis [15,16,17]. Reported bacterial etiologies include Staphylococcus aureus, Streptococcus species, pneumococcus, gram-negative bacilli, anaerobes, and mycobacteria, with variation according to geography, immune status, and preceding invasive procedures [5,18]. Although bacterial pericarditis is uncommon, delayed diagnosis can be clinically important because purulent infection may progress rapidly to tamponade, sepsis, constriction, and death.

The challenge for clinicians is therefore twofold: true bacterial pericarditis must not be missed, but indiscriminate culturing may generate false-positive or contaminant results. Such results may lead to unnecessary antimicrobial therapy, additional blood tests and imaging, infectious diseases consultations, and prolonged hospitalization. Identifying clinical and laboratory predictors of true bacterial pericarditis could improve diagnostic stewardship by focusing culture-based testing on patients with a higher probability of infection.

Accordingly, the objectives of this study were to determine the diagnostic yield of routine pericardial fluid cultures in a tertiary medical center and to identify clinical, laboratory, and imaging predictors associated with true bacterial pericarditis.

## 2. Materials and Methods

We conducted a retrospective observational and analytical study at Shaare Zedek Medical Center, a 1000-bed tertiary academic hospital in Jerusalem, Israel. The study included all pericardial fluid cultures obtained between January 2016 and May 2024. The observational component estimated the proportion of true bacterial pericarditis among patients with pericardial effusion who underwent sampling, whereas the analytical component evaluated variables associated with culture-confirmed bacterial infection.

The study protocol (0146-23-SZMC) was approved by the institutional review board and Helsinki committee of Shaare Zedek Medical center. Patient confidentiality was strictly maintained. The authors declare no conflicts of interest, and no external funding was received. All procedures were conducted in accordance with applicable ethical guidelines and regulations. The requirement for informed consent was waived because of the retrospective design of the study, use of de-identified data, absence of patient contact, and lack of any intervention related to the study.

The study population consisted of hospitalized patients for whom a pericardial fluid sample was obtained and sent for bacterial culture during the study period. At our institution, routine laboratory evaluation, including bacterial culture, is commonly performed for pericardial fluid samples. Eligible samples were identified by querying the hospital microbiology laboratory database for all specimens labeled as pericardial fluid. The medical record was then reviewed to confirm that the sample was obtained by pericardiocentesis or intra-procedural pericardial drainage. Samples were excluded when documentation suggested mislabeling or when the anatomical source could not be verified.

The primary outcome was a true positive pericardial culture, defined as growth of a recognized bacterial pathogen known to cause bacterial pericarditis or growth of an organism judged clinically pathogenic by the infectious diseases team and treated accordingly. Sterile cultures and cultures growing organisms classified as commensals according to Centers for Disease Control and Prevention criteria were considered negative or contaminants unless clinical adjudication, reported at the medical record, by infection disease specialist, supported pathogenic significance of verified the clinical course of the patient compatible with contaminate culture [19]. Because bacterial pericarditis is rare, the sample size was determined by the number of eligible cultures available during the predefined study period rather than by a priori power calculation.

Data was extracted from electronic medical records from the hospitalization during which pericardial fluid sampling was performed. Variables included demographic characteristics, comorbidities, presenting symptoms and signs, vital signs, electrocardiographic findings, chest imaging, echocardiographic features, serum laboratory values, and pericardial fluid analyses. Fluid analyses included cell count, differential, Gram stain, and acid-fast staining when available. Clinical variables were recorded only when documented before pericardial fluid culture was obtained.

Comorbidities were selected according to their potential association with pericardial effusion or infection risk. Active malignancy was defined as concurrent cancer at the time of sampling; remote cancer in remission was not included. Immunosuppression was defined broadly as any sustained condition or treatment associated with increased infection risk, including hematologic malignancy, HIV infection, or chronic immunosuppressive therapy. Short steroid courses of less than 15 days and type 2 diabetes mellitus alone were not classified as immunosuppression. Type 2 diabetes mellitus was recorded separately because it doesn’t cause direct and sustained immune compromise. Diabetes may nevertheless increase infection susceptibility and was not considered biologically irrelevant. Laboratory values and vital signs closest to the time of sampling were used. Because qSOFA and SIRS components were frequently unavailable, respiratory rate, heart rate and blood pressure in particular were analyzed instead as markers of hemodynamic status.

Categorical variables were compared using Fisher’s exact test, and continuous variables were compared using the Mann-Whitney U test. Continuous variables are reported as medians with interquartile ranges. The univariable analyses were considered exploratory and intended primarily for descriptive purposes and identification of candidate variables for multivariable analysis. Accordingly, *p*-values were not adjusted for multiple comparisons. Multivariable logistic regression was performed to identify independent predictors of true bacterial pericarditis. Two models were constructed: a full model including pericardial mononuclear cell percentage, and a trimmed model excluding this variable to maximize sample size. Odds ratios with 95% confidence intervals are reported. A two-sided *p*-value < 0.05 was considered statistically significant. Analyses were performed using SPSS software (version 31.0.0).

## 3. Results

**Figure 1:**
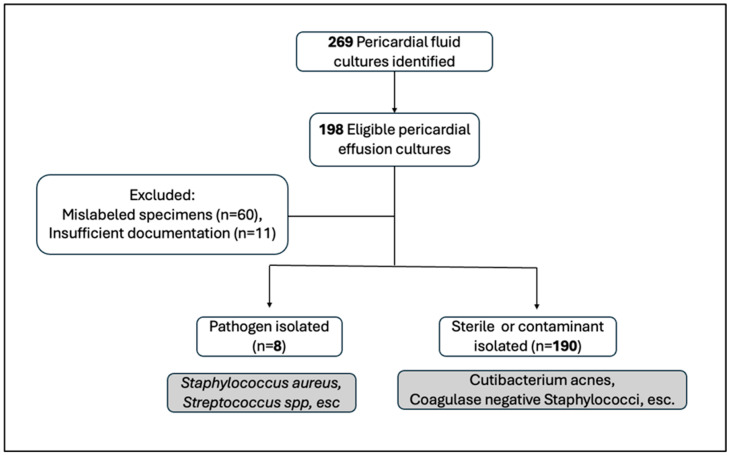
Study Flow Diagram.

A total of 269 pericardial fluid cultures were initially identified. After exclusion of mislabeled samples and cases with insufficient documentation to verify the source, 198 cultures from 188 patients were included in the final analysis (Figure 1). Eight patients contributed more than one culture during separate clinical episodes.

Overall, 8 cultures (4%) yielded true bacterial pathogens, 43 cultures (23%) grew contaminants, and 147 cultures (73%) were sterile. Because the primary objective was to identify predictors of true bacterial infection, sterile and contaminant cultures were combined for the primary regression analyses as non-pathogenic outcomes. Identified pathogens included Staphylococcus aureus (*n* = 3), Streptococcus pneumoniae (*n* = 1), Streptococcus pyogenes (*n* = 1), Escherichia coli (*n* = 1), Enterococcus faecium (*n* = 1), and two cases adjudicated as true infection despite isolation of organisms typically considered contaminants. The most frequent contaminants were Cutibacterium acnes and coagulase-negative staphylococci.

Baseline demographic characteristics, comorbidities, presenting symptoms, and vital signs did not differ significantly between patients with true positive cultures and those with negative or contaminated cultures (Table 1 and Table 2). Similarly, electrocardiographic abnormalities, chest radiography findings, effusion size, fibrinous appearance, and echocardiographic evidence of tamponade were not associated with culture positivity.

Laboratory findings are summarized in Table 3. In univariate analysis, patients with bacterial pericarditis had higher peripheral neutrophil percentages, lower lymphocyte percentages, lower platelet counts, and higher blood urea nitrogen and creatinine levels. Among pericardial fluid parameters, a lower mononuclear cell percentage was significantly associated with bacterial infection.

In multivariable analysis including pericardial mononuclear cell percentage, both lower mononuclear percentage and higher peripheral neutrophil percentage independently predicted bacterial pericarditis (Table 4). In the trimmed model excluding pericardial cell data, concurrent bacteremia and peripheral neutrophilia remained independently associated with true culture positivity. Because only eight true-positive cultures were identified, the multivariable analyses are at substantial risk of overfitting and imprecise effect estimation.

## 4. Discussion

In this large single-center cohort spanning more than eight years, bacterial pericarditis was uncommon, accounting for only 4% of pericardial fluid cultures. In contrast, nearly one quarter of cultures yielded organisms classified as contaminants. These findings highlight the limited diagnostic efficiency of routine culturing when applied broadly to all pericardial fluid samples, regardless of clinical suspicion.

Most cultures in our cohort were sterile. A sterile culture may represent truly noninfectious or nonbacterial pericardial fluid, which is expected in many patients with malignant, inflammatory, uremic, traumatic, or idiopathic effusions. However, false-negative results are also possible. Molecular methods such as polymerase chain reaction (PCR), used in addition to conventional culture, are recognized as important diagnostic tools when cultures are negative or when organisms are difficult to isolate. This is particularly relevant for viral pathogens, Mycobacterium tuberculosis, and fastidious bacteria [7,20,21]. Our medical records did not consistently include PCR results for culture-negative cases, and we therefore could not determine how often molecular testing would have changed the final etiologic classification.

Another possible explanation for sterile cultures is prior admission antibiotic treatment. Evidence from pleural effusion studies indicates that antibiotic exposure before sampling reduces culture yield. In a Danish population-based study of 840 pleural fluid specimens, multivariable analysis demonstrated an inverse association between the duration of prior antibiotic therapy and pleural fluid culture positivity [22]. Direct evidence for pericardial fluid is more limited, but the same biological principle is plausible because antimicrobial exposure may suppress viable organisms while inflammatory findings persist. The observed 4% rate therefore represents the proportion of cultures yielding a confirmed bacterial pathogen, not necessarily the prevalence of all bacterial pericardial infections in the sampled cohort.

Pericardial-specific studies further support the potential value of molecular methods. In a 2006 study of 106 pericardial fluid samples, four bacterial infections were not detected by conventional culture after prior antibiotic exposure, including Mycobacterium tuberculosis, Streptococcus pneumoniae, and Actinomyces neuii; these cases were identified only by molecular techniques [20]. Similarly, a 2025 study evaluating Molecular Culture ID, a PCR-based pan-bacterial assay, in 42 pericardial effusions found more positive results by molecular testing than by conventional culture [21]. When bacterial pericarditis is clinically suspected and antibiotics have already been initiated, PCR-based assays should therefore be considered because they may identify pathogens missed by culture.

Our findings are consistent with prior reports showing that bacterial pericarditis represents a small minority of pericardial effusions [15,16,17]. Importantly, commonly relied upon clinical features, including fever, chest pain, dyspnea, and echocardiographic tamponade, did not reliably distinguish bacterial from nonbacterial etiologies in our cohort. This may reflect the nonspecific nature of systemic inflammatory symptoms in hospitalized patients and the fact that tamponade physiology is determined more by the rate and volume of fluid accumulation than by the underlying microbiologic cause [23,24].

The absence of discriminatory echocardiographic findings should not be interpreted as evidence that imaging lacks clinical value. Echocardiography remains essential for detecting and quantifying effusion, assessing hemodynamic consequences, and guiding drainage. However, its ability to establish microbiological etiology is limited. More broadly, Angeli et al., in a prospective cohort of 142 patients evaluated for cardiac masses, reported that echocardiography missed 12.7% of lesions, particularly those involving the pericardium and great vessels, and demonstrated that even multimodality imaging may yield overlapping appearances among malignant, inflammatory, and infectious processes [25]. Although that population differs from ours, the findings reinforce the principle that imaging findings alone cannot reliably establish or exclude the microbiological cause of pericardial disease.

Concurrent bacteremia emerged as the strongest predictor of bacterial pericarditis—with the limitation of the small number of true bacteremia cultures, which could lead to the results being overinterpreted—supporting hematogenous spread of the microorganism into the pericardial space as an important pathogenic mechanism. Peripheral neutrophilia also independently predicted infection, reflecting activation of the innate immune response to bacterial infection, with cytokine-mediated mobilization of circulating neutrophils and recruitment of polymorphonuclear leukocytes resulting in systemic neutrophilia and neutrophil predominance within infected tissues. In patients with available pericardial fluid cell counts, a lower mononuclear cell percentage was additionally associated with true infection, consistent with a predominantly neutrophilic inflammatory response. Together, these findings suggest that laboratory-based criteria may provide greater diagnostic discrimination than symptoms or imaging alone.

Certain populations may also warrant targeted testing despite the low overall yield of routine cultures. The literature identifies groups at increased risk for tuberculous pericarditis, including patients living in or originating from tuberculosis-endemic regions, unhoused individuals, people with current or prior incarceration, and immunocompromised patients, including those with HIV infection [26,27]. These variables were not consistently available in our dataset, limiting our ability to assess their contribution. In clinical practice, however, epidemiologic risk should remain an important component of the decision to send mycobacterial cultures, acid-fast staining, adenosine deaminase testing, or molecular assays.

The high contaminant rate observed in this study has practical implications. False-positive cultures can trigger unnecessary antimicrobial therapy, repeat blood cultures, additional imaging, prolonged observation, and patient anxiety [28]. They may also complicate interpretation in patients with multiple comorbidities or indwelling devices, in whom distinguishing contamination from true infection can be challenging. A more selective strategy, informed by bacteremia, peripheral neutrophilia, pericardial fluid differential, and epidemiologic risk, may improve the balance between diagnostic sensitivity and stewardship.

Unlike blood cultures, for which contamination rates are relatively well established and depend on collection technique, contamination rates for pericardial and pleural fluid cultures are not systematically reported [22,29]. One potential source of contamination is catheter-drawn sampling. During pericardiocentesis, pericardial fluid is commonly obtained through a catheter, which may introduce skin flora or other commensal organisms into the specimen. Therefore, catheter-based collection may have contributed to some of the contaminant cultures observed in our cohort.

The procedural setting may also influence contamination risk. In our center, nearly all pericardiocentesis procedures are performed in the catheterization laboratory under sterile conditions. Procedures performed at the bedside may carry a higher contamination risk because of differences in environmental control, urgency, operator conditions, and sterility logistics [30]. However, the procedural location was not consistently documented in our dataset. We could not therefore assess whether sampling site contributed to contamination, and this missing information represents a relevant limitation.

Several limitations merit consideration. The retrospective design introduces potential selection and documentation bias. The single-center setting may limit generalizability, particularly to regions with substantially different tuberculosis prevalence or different microbiology laboratory practices. Pericardial cell counts were unavailable for a subset of patients, necessitating a reduced multivariable model. The small number of true positive cases limited statistical power and widened confidence intervals. Another limitation is that complete-case analysis may introduce selection bias if the availability of pericardial cell counts was associated with disease severity or other clinical characteristics. Finally, data regarding prior admission antibiotic exposure, culture timing related to symptoms, volume of pericardial fluid, molecular testing, and procedure location were incomplete, limiting our ability to fully evaluate causes of culture-negative infection or contamination.

Despite these limitations, the study has several strengths. It includes an extended study period, a relatively large number of pericardial fluid cultures for a rare clinical condition, comprehensive chart review, adjudication of microbiologic findings using clinical context, and complementary regression models. These features support the clinical relevance of the findings and provide a practical basis for prospective validation.

## 5. Conclusions

Bacterial pericarditis was rare in this cohort, and routine pericardial fluid cultures had low diagnostic yield with substantial contamination. Concurrent bacteremia, peripheral neutrophilia, and, when available, a low percentage of pericardial fluid mononuclear cells may help identify patients most likely to benefit from bacterial culture. Selective, laboratory-guided testing, supplemented by molecular assays in appropriate clinical contexts, may improve diagnostic efficiency and reduce unnecessary downstream interventions. Prospective multicenter studies are warranted to validate these predictors and inform evidence-based diagnostic strategies.

## Figures and Tables

**Table 1:** Demographic Characteristics of Patients with Pericardial Fluid Culture.

Characteristic	All (*N* = 198)	True-Positive Culture (*N* = 8)	Negative Culture or Contamination (*N* = 190)	*p* Value
**Gender,** **no. (%)**				
Male	128 (64.6)	6 (75)	122 (64.2)	0.72
Female	70 (35.4)	2 (25)	68 (35.8)	
Age, years, median (IQR)	64 (46–75)	67.5 (46–74)	63.5	0.5
**Background conditions, no. (%)**				
Malignancy	39 (19.7)	0 (0)	39 (20.5)	0.36
Autoimmunity	24 (12.1)	0 (0)	24 (12.6)	0.6
ESRD and dialysis	4 (2)	0 (0)	4 (2.1)	1
Immunosuppression	17 (8.6)	0 (0)	17 (8.9)	1
**Concurrent infections and possible sources, no. (%)**				
Bacteremia	10 (5)	4 (50)	6 (3.2)	<0.001
Endocarditis	3 (1.5)	1 (12.5)	2 (1.1)	0.12
Pulmonary infection	19 (9.6)	1 (12.5)	18 (9.5)	0.56
CNS infection	3 (1.5)	1 (12.5)	2 (1.1)	0.12
Penetrating chest trauma	37 (18.7)	1 (12.5)	36 (18.9)	1
Other	10 (5.1)	2 (25)	8 (4.2)	0.06

Abbreviations: CNS, central nervous system; ESRD, end-stage renal disease; IQR, interquartile range.

**Table 2:** Clinical Characteristics of Patients with Pericardial Fluid Culture.

Characteristic	All (*N* = 198)	True-Positive Culture (*N* = 8)	Negative Culture or Contamination (*N* = 190)	*p* Value
**Symptoms & signs, no. (%)**				
Fever	32 (16.5)	2 (25)	30 (16.1)	0.62
Dyspnea	126 (63.6)	5 (62.5)	121 (63.7)	1
Chest pain	80 (40.4)	3 (37.5)	77 (40.5)	1
Tachycardia	62 (31.5)	4 (50)	58 (30.7)	0.26
Cough	63 (26.8)	2 (25)	51 (28.8)	1
Sore throat	8 (4)	0 (0)	8 (4.2)	1
GI symptoms	25 (12.6)	1 (12.5)	24 (12.6)	1
**Vital signs, median (IQR)**				
Pulse, bpm	90 (74–106)	98 (78–111)	90 (73–105)	0.44
Systolic blood pressure, mmHg	115 (100–132)	108 (93–122)	115 (101–133)	0.21
Diastolic blood pressure, mmHg	74 (64–80)	76 (64–89)	73 (64–80)	0.522
**Physical exam and imaging, no./total no. (%)**				
Pericardial rub	11/198 (5.6)	0/8 (0)	11/190 (5.8)	1
ECG abnormalities	52/168 (31)	1/6 (16.7)	51/162 (31.5)	0.67
Chest X-ray abnormalities	103/171 (60.2)	3/6 (50)	100/165 (60.6)	0.68
Effusion size by echocardiography				0.66
Small	4/187 (2.1)	0/5 (0)	4/182 (2.2)	
Medium	52/187 (27.8)	2/5 (40)	50/182 (27.5)	
Large	131/187 (70.1)	3/5 (60)	128/182 (70.3)	
Fibrin in effusion	20/187 (10.7)	0/5 (0)	20/182 (11)	1
Tamponade signs by echo	59/193 (30.6)	2/7 (28.6)	57/186 (30.6)	1

Abbreviations: ECG, electrocardiogram; GI, gastrointestinal; IQR, interquartile range.

**Table 3:** Blood and pericardial fluid laboratory results.

Laboratory test	True-Positive Culture (*N* = 8)	Negative Culture or Contamination (*N* = 190)	*p* Value
Bacteremia	4 (50)	6 (3.2)	<0.001
**Blood specimen, median (IQR)**			
Leukocytes (10^3^/μL)	11.3 (8.1–14.7)	9.6 (7.3–12.8)	0.582
Neutrophiles (%)	86.9 (83.6–88.7)	75.4 (68.3–81)	<0.0001
Lymphocytes (%)	6.9 (4.8–8.6)	13.7 (8.3–19.8)	0.001
Platelets (10^3^/μL)	178 (152–208)	276 (223–356)	0.016
Glucose (mg/dL)	159 (85–187)	113 (95–136)	0.357
BUN (mg/dL)	43.5 (31.5–82.3)	18 (13–32)	0.001
Creatinine (mg/dL)	1.32 (1.16–2.94)	0.9 (0.71–1.2)	0.021
CRP (mg/dL)	6.82 (5.04–42.87)	6.99 (2.14–14.18)	0.158
TSH (mIU/L)	2.02 (1.79–3.03)	1.96 (1.12–3.01)	0.717
pH	7.41 (7.34–7.45)	7.40 (7.36–7.44)	0.920
**Pericardial fluid specimen-median (IQR)**			
Glucose (mg/dL)	48 (16–93)	86 (69–115)	0.243
Protein	4.05 (3.6–4.71)	5.14 (4.42–5.7)	0.176
Leukocytes (cell/μL)	7111 (3647–29,146)	2374 (728–4449)	0.125
Mononuclears (%)	6.8 (3.7–14.2)	59.2 (37.6–80.9)	0.003
Polymorphonuclears (%)	85.8 (54.1–93.2)	40.8 (19.1–62.4)	0.144

Abbreviations: BUN, blood urea nitrogen; CRP, C-reactive protein; IQR, interquartile range; TSH, thyroid-stimulating hormone.

**Table 4:** Blood and pericardial fluid stepwise multivariable regression model.

Laboratory Test	Multivariable Logistic Regression of True Positive Pericardial Culture	
	Full Analysis (*N* = 133) Adjusted OR (95% CI), *p*-Value	Excluding Mononuclears in Pericardial Fluid (*N* = 196) Adjusted OR (95% CI), *p* Value
Bacteremia	Not retained, 0.69	17.49 (2.9–103.5), 0.002
**Blood specimen, median (IQR)**		
Neutrophiles (%)	1.36 (1.03–1.79), 0.03	1.2 (1.04–1.39), 0.012
Lymphocytes (%)	Not retained, 0.36	Not retained, 0.94
Platelets (10^3^/μL)	Not retained, 0.18	Not retained, 0.65
BUN (mg/dL)	Not retained, 0.94	Not retained. 0.35
Creatinine (mg/dL)	Not retained, 0.384	Not retained. 0.56
**Pericardial fluid specimen-median (IQR)**		
Mononuclear (%)	0.91 (0.83–0.99), 0.03	

Adjusted odds ratios were calculated using multivariable logistic regression. Candidate covariates were bacteremia, peripheral neutrophil percentage, lymphocyte percentage, platelet count, blood urea nitrogen, creatinine, and pericardial-fluid mononuclear-cell percentage. The full model included pericardial-fluid mononuclear-cell percentage; the reduced model excluded this variable because of missing data. Variables not retained during stepwise selection are reported with the probability associated with removal from the model. Odds ratios are shown only for variables retained in the final model.

## Data Availability

The data presented in this study are available on request from the corresponding author due to privacy and ethical restrictions.

## Abbreviations

The following abbreviations are used in this manuscript:

CDC	Center Disease Control
